# The CARE guidelines: consensus-based clinical case reporting guideline development

**DOI:** 10.1186/1752-1947-7-223

**Published:** 2013-09-10

**Authors:** Joel J Gagnier, Gunver Kienle, Douglas G Altman, David Moher, Harold Sox, David Riley

**Affiliations:** 1 Department of Orthopaedic Surgery, University of Michigan, Ann Arbor, MI, USA; 2 Department of Epidemiology, School of Public Health, University of Michigan, Ann Arbor, MI, USA; 3 Institute for Applied Epistemology and Medical Methodology, University of Witten/Herdecke, Freiburg, Germany; 4 Centre for Statistics in Medicine, University of Oxford, Oxford, UK; 5 Ottawa Hospital Research Institute, Ottawa, Canada; 6 Department of Epidemiology and Community Medicine, University of Ottawa, Ottawa, ON, Canada; 7 The Dartmouth Institute and Geisel School of Medicine at Dartmouth, Hannover, NH, USA; 8 Global Advances in Health and Medicine, Portland, OR, USA; 9 For a complete list of the CARE Group see the Author Contributions

**Keywords:** Case report, Case study, EQUATOR Network, Patient reports, Meaningful use, Health research reporting guidelines

## Abstract

**Background:**

A case report is a narrative that describes, for medical, scientific, or educational purposes, a medical problem experienced by one or more patients. Case reports written without guidance from reporting standards are insufficiently rigorous to guide clinical practice or to inform clinical study design.

*Primary Objective.* Develop, disseminate, and implement systematic reporting guidelines for case reports.

**Methods:**

We used a three-phase consensus process consisting of (1) pre-meeting literature review and interviews to generate items for the reporting guidelines, (2) a face-to-face consensus meeting to draft the reporting guidelines, and (3) post-meeting feedback, review, and pilot testing, followed by finalization of the case report guidelines.

**Results:**

This consensus process involved 27 participants and resulted in a 13-item checklist—a reporting guideline for case reports. The primary items of the checklist are title, key words, abstract, introduction, patient information, clinical findings, timeline, diagnostic assessment, therapeutic interventions, follow-up and outcomes, discussion, patient perspective, and informed consent.

**Conclusions:**

We believe the implementation of the CARE (CAse REport) guidelines by medical journals will improve the completeness and transparency of published case reports and that the systematic aggregation of information from case reports will inform clinical study design, provide early signals of effectiveness and harms, and improve healthcare delivery.

## Introduction

### A case report is a detailed narrative that describes, for medical, scientific, or educational purposes, a medical problem experienced by one or several patients

Case reports present clinical observations customarily collected in healthcare delivery settings. They have proved helpful in the identification of adverse and beneficial effects, the recognition of new diseases, unusual forms of common diseases, and the presentation of rare diseases [1]. For example, our understanding of the relationship between thalidomide and congenital abnormalities [2] and the use of propranolol for the treatment of infantile hemangiomas began with case reports [3]. Case reports may generate hypotheses for future clinical studies, prove useful in the evaluation of global convergences of systems-oriented approaches, and guide the individualization and personalization of treatments in clinical practice [4,5]. Furthermore, case reports offer a structure for case-based learning in healthcare education and may facilitate the comparison of healthcare education and delivery across cultures.

Case reports are common and account for a growing number of articles in medical journals [6]; however their quality is uneven [7,8]. For example, one study evaluated 1316 case reports from four peer-reviewed emergency-medicine journals and found that more than half failed to provide information related to the primary treatment that would have increased transparency and replication [9]. Written without the benefit of reporting guidelines, case reports often are insufficiently rigorous to be aggregated for data analysis, inform research design, or guide clinical practice [7,9].

Reporting guidelines exist for a variety of study designs including randomized controlled trials (Consolidated Standards of Reporting Trials, or CONSORT) [10], observational studies (Strengthening the Reporting of Observational studies in Epidemiology, or STROBE) [11], and systematic reviews and meta-analyses (Preferred Reporting Items for Systematic Reviews and Meta-Analyses, or PRISMA) [12]. Empirical evidence suggests that a journal’s adoption of the CONSORT statement as a guide to authors is associated with an increase in the completeness of published randomized trials [13]. Guidelines have been developed for adverse-event case reports [14]; however, general reporting guidelines for case reports do not exist. Our primary objective was to develop reporting guidelines for case reports through a consensus-based process.

## Methods

### Research design

We followed the Guidance for Developers of Health Research Reporting Guidelines [15] and developed a three phase consensus process [16]. This consisted of (1) a pre-meeting literature review followed by interviews to generate items for a case report checklist, (2) a face-to-face consensus meeting for drafting a reporting guideline, and (3) post-meeting feedback and pilot testing followed by finalization of the case report guidelines.

### Participants

We contacted 28 individuals who fulfilled at least one of four criteria [17-19]: (1) publication of articles related to case reports; (2) publication of a manual, handbook, or method guidelines related to case reports; (3) publication of a systematic review of methods or reporting related to case reports; and (4) publication of other reporting guidelines for clinical research.

### Consensus process

Phase 1: Four of the authors, the steering committee (JG, GK, DM, and DR), searched the literature for publications on the role of case reports, recommendations for their publication, and surveys on reporting quality. A letter was sent to 28 potential participants explaining the purpose of the meeting, details of the consensus technique, and requesting their participation in generating specific recommendations for case reporting. Twenty-seven people agreed to participate and were scheduled for a telephone interview and sent a selection of key articles on case reports. During the telephone interview, participants were asked (1) what information was required to be included in case-reporting guidelines, (2) the rationale for their suggestions, and (3) for references that supported their reasoning.

Three of the authors (JG, GK, and DR) grouped the recommendations from the literature search and interviews by theme together with their rationale, references, and operational definitions. No quantitative scoring was done.

Phase 2: The face-to-face consensus meeting at the University of Michigan in Ann Arbor (October 2012) included 18 participants from Phase 1, one research assistant and two student observers. The meeting began with a review of the blinded recommendations elicited during the Phase 1 interviews, in whole group and small group sessions. On the second day, open discussion of each potential item continued, during which clarifications, opinions, justifications, operational definitions, and new ideas were expressed. By the end of the second day, the group had agreed upon a set of preliminary reporting recommendations.

Phase 3: The draft checklist was refined by the steering committee and sent for two rounds of review to the complete group (Phase 1 and 2 participants). The finalized reporting guidelines incorporated the feedback from the entire CARE group.

## Results

The CAse REport (CARE) guidelines checklist is structured to correspond with key components of a case report and capture useful clinical information (including ‘meaningful use’ information mandated by some insurance plans).

The checklist begins with a statement that describes the narrative of a case report. The meeting CARE group felt that a case report should tell a story using prose that has a consistent style across all sections, including the rationale for any conclusions and take-away messages.

We recommend a timeline (item 7) in the form of a table or figure that gives the specific dates and times of important components of the case. This might include family and past medical history, genetic information, current symptoms, diagnostic test results, interventions, and events that occurred during follow-up. The timeline should show how the key events of the case unfolded.

We created separate checklist items for diagnostic assessments (item 8) and therapeutic interventions (item 9) with the recognition that both items will often be relevant in a case report.

The group discussed at length whether to include the patient’s perspective on his or her experience. In the end, we advocated for patient-reported outcomes and experiences whenever possible (item 12). There was also discussion about the need for guidelines for patient-reported outcomes of their care. In a similar vein, a recent extension of the CONSORT statement was published for patient-reported outcomes in randomized trials; CONSORT-PRO [20].

Finally, we included an item on informed consent (item 13). We believe that authors have an ethical duty to obtain informed consent from the patient to publish patient information in a case report. Consent becomes informed when the patient or a relative reads the case report and approves its contents. If the patient cannot give consent and attempts to find a relative to give proxy consent have failed, the authors should seek permission to publish from an institutional committee. There may be other circumstances where an ethics committee or Institutional Review Board (IRB) approval may be necessary. The CARE guidelines are shown in the following Table 1.

**Table 1:** The CARE guidelines checklist

**The Narrative:** A case report tells a story in a narrative format that includes the presenting concerns, clinical findings, diagnoses, interventions, outcomes (including adverse events), and follow-up. The narrative should include a discussion of the rationale for any conclusions and any take-away messages.		
**Item name**	**Item no.**	**Brief description**
**Title**	1	The words “case report” (or “case study”) should appear in the title along with phenomenon of greatest interest (eg, symptom, diagnosis, test, intervention)
**Keywords**	2	The key elements of this case in 2-5 words
**Abstract**	3	a) Introduction—What does this case add?
		b) Case Presentation:
		- The main symptoms of the patient
		- The main clinical findings
		- The main diagnoses and interventions
		- The main outcomes
		c) Conclusion—What were the main “take-away” lessons from this case?
**Introduction**	4	Brief background summary of this case referencing the relevant medical literature
**Patient Information**	5	a) Demographic information (eg, age, gender, ethnicity, occupation)
		b) Main symptoms of the patient (his or her chief complaints)
		c) Medical, family, and psychosocial history—including diet, lifestyle, and genetic information whenever possible, and details about relevant comorbidities including past interventions and their outcomes
**Clinical Findings**	6	Describe the relevant physical examination (PE) findings
**Timeline**	7	Depict important dates and times in this case (table or figure)
**Diagnostic Assessment**	8	a) Diagnostic methods (eg, PE, laboratory testing, imaging, questionnaires)
		b) Diagnostic challenges (eg, financial, language/cultural)
		c) Diagnostic reasoning including other diagnoses considered
		d) Prognostic characteristics (eg, staging) where applicable
**Therapeutic Intervention**	9	a) Types of intervention (eg, pharmacologic, surgical, preventive, self-care)
		- Administration of intervention (eg, dosage, strength, duration)
		- Changes in intervention (with rationale)
**Follow-up and Outcomes**	10	a) Summarize the clinical course of all follow-up visits including
		- Clinician and patient-assessed outcomes
		- Important follow-up test results (positive or negative)
		- Intervention adherence and tolerability (and how this was assessed)
		- Adverse and unanticipated events
**Discussion**	11	a) The strengths and limitations of the management of this case
		b) The relevant medical literature
		c) The rationale for conclusions (including assessments of cause and effect)
		d) The main “take-away” lessons of this case report
**Patient Perspective**	12	The patient should share his or her perspective or experience whenever possible
**Informed Consent**	13	Did the patient give informed consent? Please provide if requested

## Discussion

This 13-item checklist provides a framework to satisfy the need for completeness and transparency for published case reports. We attempted to strike a balance between adequate detail and the concise writing that is one of the appealing characteristics of a case report. Our consensus process resulted in a set of essential items for authors to consider when submitting a case report for publication.

While case reports have long been an important source of new ideas and information in medicine [21], it appears that case reports are likely to begin to play a role in the discovery of what works and for whom. BioMed Central launched the *Journal of Medical Case Reports* in 2007 [22] and a Cases Database in 2012 with more than 11,000 published case reports from 50 medical journals. In 6 months, it has grown to more than 26,000 case reports from 212 medical journals [23]. The CARE guidelines checklist is part of a growing effort to improve the reporting of case reports.

There is substantial empirical evidence that reporting guidelines improve the completeness of published scientific reports eg, see references [13,24,25]. A recent Cochrane review examining the influence of journal endorsement of the CONSORT statement on reporting included 53 publications assessing 16,604 randomized controlled trials and found that CONSORT-endorsing journals consistently have better overall reporting [13]. However, the potential impact of the CONSORT statement and related reporting guidelines has not been fully realized. A study examining the instructions to peer reviewers of 116 health research journals found that only 41 (35%) provided online instructions to peer reviewers. Of those, only 19 (46%) mentioned or referred to reporting guidelines as a useful resource [26]. In response, the authors provide several recommendations for editors to improve the peer review of submitted manuscripts, suggesting that journals have a responsibility to support peer reviewers [26].

The developers of reporting guidelines have a responsibility to plan a dissemination and implementation strategy that supports guidelines utilization [15]. Our efforts have several components:

1. The CARE guidelines will be presented at international conferences and workshops including the Peer Review and Biomedical Publication Congress in Chicago on September 10, 2013.

2. This article will be published simultaneously in multiple medical journals and outreach to the 212 journals depositing case reports into the BioMed Central Case Report Database.

3. We will develop a more detailed explanation and elaboration article to outline the rationale for each item and include empirical evidence and examples of good reporting from published case reports.

4. The CARE guidelines are being pilot tested, and preliminary results support the guidelines as currently written (personal communication with Helmut Kiene, Erika Oberg, Bill Manahan). Guidelines extensions for specialties are being developed.

5. The CARE guidelines and related documents will be available on a dedicated website (www.CARE-statement.org), the EQUATOR Network website (www.equator-network.org), and translated into multiple languages.

6. Authors, journal editors, peer reviewers and the wider medical community are encouraged to use the CARE checklist and provide feedback that can be incorporated into regular updates of the CARE guidelines.

7. We will conduct and support research into the impact of the CARE guidelines on the reporting of case reports.

### Limitations

The CARE guidelines and their development have several possible limitations. First, these guidelines were developed through a consensus method and thus represent the opinions of the participants. However, consensus was easily reached during our meeting, we referred to the empirical evidence where available, and we received feedback from a wide selection of individuals, beyond those involved in our consensus meeting. Second, we recognize that causality determinations are a challenge for case reports even when following reporting guidelines [27,28]. The CARE guidelines emphasize information quality independent of causality assessments. Different specialties, practitioners, and patients are likely to require extensions of the CARE guidelines with specialty specific information. We welcome discussions with groups interested in using the CARE guidelines as the basis for their specific reporting needs.

Though not mentioned in our guidelines, medical journals often require authors to address three issues: (a) potential competing interests, (b) de-identification of patient-related data, and (c) ethics committee or Institutional Review Board (IRB) approval if obtained or necessary.

## Conclusions

Anticipating a long future for case reports, we have provided guidance in the form of reporting standards for use by healthcare stakeholders around the world. The growth of case reports in an era in which clinical trials and systematic reviews dominate the tables of content of medical journals indicates that case reports have value, particularly with the increasing importance of individualized care. Unlike randomized controlled trials, case reports are individual reports related to the care of individual patients where the sample size is one. When systematically collected and combined into larger datasets, they can be analyzed, enhancing the early discovery of effectiveness and harms.

We anticipate that the analysis of systematically aggregated information from patient encounters (now mandated by some insurance plans) will provide scalable, data-driven insights into what works for which patients in real time, facilitating comparisons across medical systems and cultures. Practitioners will soon be able to provide—and in some cases they are required to provide—patients with information from their encounters. This will transform how we think about “evidence” and revolutionize its creation, diffusion, and use—opening new opportunity landscapes. When it becomes clear how new data contributes to evidence, the stewardship needed to produce high-quality data will be more rewarding and our attitude toward “observation” will shift. The CARE guidelines provide a framework to satisfy the need for precision, completeness, and transparency.

## Author contributions

JG, GK, DGA, DM, HS, and DR met the ICMJE criteria for authorship. JG and DR wrote the first draft of the article. DGA, JG, GK, DM, DR, and HS critically reviewed and edited drafts. The entire CARE group participated in parts or all of the guidelines development process and contributed to the editing and revision of the CARE guidelines and this article. All authors read and approved the final manuscript.

### The CARE group

1. Alyshia Allaire, BA, Portland, OR, USA

2. Douglas G. Altman, DSc, Centre for Statistics in Medicine, University of Oxford, Oxford, UK

3. Jeffrey Aronson, MB, ChB, MA Dphil, FRCP, FB, PharmacolS, University of Oxford, Oxford, UK*

4. James Carpenter, MD, Department of Orthopaedic Surgery, University of Michigan, Ann Arbor, MI, USA

5. Joel Gagnier, ND, MSc, PhD, Departments of Orthopaedic Surgery and Epidemiology, University of Michigan, Ann Arbor, MI, USA

6. Patrick Hanaway, MD, Director of Medical Education, Institute for Functional Medicine, Asheville, NC, USA*

7. Carolyn Hayes, PhD, RN, NEA-BC, Dana-Farber Brigham and Women’s Cancer Center, Boston, MA, USA

8. David Jones, MD, President, Institute for Functional Medicine, Ashland, OR, USA

9. Marietta Kaszkin-Bettag, PhD, University of Frankfurt, Pharmalex GmbH, Mannheim, Germany

10. Michael Kidd, AM, MD, Editor-in-Chief *Journal of Medical Case Reports*, Faculty of Health Sciences, Flinders University, Adelaide, Australia*

11. Helmut Kiene, Dr med, Editor, *Global Advances in Health and Medicine,* Institute for Applied Epistemology and Research Methodology, University of Witten/Herdecke, Freiburg, Germany

12. Gunver Kienle, Dr med, Editor, *Global Advances in Health and Medicine,* Institute for Applied Epistemology and Research Methodology, University of Witten/Herdecke, Freiburg, Germany

13. Ben Kligler, MD, MPH, Co-Editor-in-Chief *Explore*, Beth Israel Medicine Center, New York, NY, USA*

14. Lori Knutson, RN, BSN, HN-BC, Integrative Healthcare Solutions, Minneapolis, MN, USA

15. Christian A. Koch, MD, PhD, FACP, FACE, Deputy Editor *Journal of Medical Case Reports,* University of Mississippi, Jackson, MS, USA*

16. Karen Milgate, MPP, Independent Health Policy Consultant, Washington, DC, USA*

17. Michele Mittelman, RN, MPH, Editor, *Global Advances in Health and Medicine*, Dover, MA, USA

18. David Moher, PhD, Ottawa Hospital Research Institute; Department of Epidemiology and Community Medicine, University of Ottawa, Ottawa, ON Canada*

19. Hanna Oltean, MPH, University of Michigan, Ann Arbor, MI, USA

20. Greg Plotnikoff, MD, MTS, FACP, Editor, *Global Advances in Health and Medicine,* Allina Center for Healthcare Innovations and the Penny George Institute for Health and Healing, Minneapolis, MN, USA

21. Richard Alan Rison, MD, FAANEM, Deputy Editor, *Journal of Medical Case Reports,* Section Editor, *BMC Research Notes*, PIH Health Hospital, Whittier, University of Southern California, Los Angeles, CA, USA*

22. David Riley MD, Editor-in-Chief, *Global Advances in Health and Medicine*, Portland, OR, USA

23. Anil Sethi, MS, Johns Hopkins School of Medicine - Information architecture and IT, Palo Alto, CA, USA*

24. Larissa Shamseer, MSc, Ottawa Hospital Research Institute, Ottawa, ON, Canada

25. Richard Smith, MB, ChB, MSc, United Healthcare Chronic Disease Initiative, London, UK

26. Harold Sox, MD, The Dartmouth Institute, Hannover, NH, USA

27. Peter Tugwell, MD, FRCP, University of Ottawa, Ottawa, ON, Canada

* Participated in the guidelines development process, the review and editing of the CARE guidelines and this article, but did not attend the face-to-face consensus meeting.
